# Early administration of multiple vasopressors is associated with better survival in patients with sepsis: a propensity score-weighted study

**DOI:** 10.1186/s40001-023-01229-w

**Published:** 2023-07-22

**Authors:** Xin Tong, Xiaopeng Xue, Chuanzhi Duan, Aihua Liu

**Affiliations:** 1 Beijing Neurosurgical Institute, Beijing Tiantan Hospital, Capital Medical University, Beijing, 100070 China; 2 Department of Cerebrovascular Surgery, Engineering Technology Research Center of Education Ministry of China on Diagnosis and Treatment of Cerebrovascular Disease, Zhujiang Hospital, Neurosurgery Center, Southern Medical University, Guangdong, China; 3 Guangdong Provincial Key Laboratory on Brain Function Repair and Regeneration, Guangdong, China

**Keywords:** Sepsis, Vasopressor, Multiple agents, Mortality, Early treatment

## Abstract

**Background:**

The association between the timing of administration of multiple vasopressors and patient outcomes has not been investigated.

**Methods:**

This study used data from the MIMIC-IV database. Patients with sepsis who were administered two or more vasopressors were included. The principal exposure was the last norepinephrine dose when adding a second vasopressor. The cohort was divided into early (last norepinephrine dose < 0.25 μg/kg/min) and normal (last norepinephrine dose ≥ 0.25 μg/kg/min) groups. The primary outcome was 28-day mortality. Multivariable Cox analyses, propensity score matching, stabilized inverse probability of treatment weighting (sIPTW), and restricted cubic spline (RCS) curves were used.

**Results:**

Overall, 1,437 patients who received multiple vasopressors were included. Patients in the early group had lower 28-day mortality (HR: 0.76; 95% CI: 0.65–0.89; p < 0.001) than those in the single group, with similar results in the propensity score-matched (HR: 0.80; 95% CI: 0.68–0.94; p = 0.006) and sIPTW (HR: 0.75; 95% CI: 0.63–0.88; p < 0.001) cohorts. RCS curves showed that the risk of 28-day mortality increased as the last norepinephrine dose increased.

**Conclusions:**

The timing of secondary vasopressor administration is strongly associated with the outcomes of patients with sepsis.

**Supplementary Information:**

The online version contains supplementary material available at 10.1186/s40001-023-01229-w.

## Background

Vasodilatory shock is the most prevalent type of shock, accounting for almost two-thirds of all cases [1–3]. As vasodilatory shock leads to insufficient delivery of oxygen to tissues and end-organ dysfunction, fluid administration and vasopressors are necessary to rectify hypotension and low blood flow [1, 4]. Previously, vasodilatory shock was treated using a stepwise method based on the blood pressure (BP) using fluid administration, vasopressor administration, and lastly, increased doses of or additional vasopressors. However, this classical method is currently being challenged as it may delay the timing of sufficient perfusion, resulting in poor outcomes in patients with refractory septic shock [5–7]. In these patients, the early administration of vasopressors to establish adequate perfusion pressure is critical to improving outcomes [8]. However, the choice and timing of vasopressors remain controversial [5, 9–11].

Norepinephrine (NE) has been the first-line treatment for vasodilatory shock. The Surviving Sepsis Campaign (SSC) recommends adding a second vasopressor (such as vasopressin) when patients have an inadequate mean BP after the administration of 0.25−0.5 μg/kg/min NE rather than increasing its dose [1]. Additionally, the authors of another study recommend the early application of a multimodal vasopressor treatment strategy in patients with complicated vasodilatory shock [5]. Similar to the early administration of broad-spectrum antimicrobials in patients with suspected and confirmed sepsis, the use of broad-spectrum vasopressors may improve outcomes in patients with refractory vasodilatory shock [12]. However, the optimal timing of administration of multiple vasopressors has not yet been established, leading to significant variations in the clinical application of multiple vasopressors [13].

Therefore, this study investigated the relationship between the timing of administration of multiple vasopressors and outcomes in patients with sepsis, as well as whether early vasopressor administration is associated with improved clinical outcomes, to better understand the benefits of the use of several vasopressors in the management of septic shock.

## Methods

### Study population

This retrospective observational study used data from the MIMIC-IV database, which contains de-identified health-related data from over 40,000 unique patients who were treated in critical care units of the Beth Israel Deaconess Medical Center between 2008 and 2019 [14]. The database is publicly available on PhysioNet [15] (https://physionet.org/content/mimiciv/2.0/). The MIMIC-IV database has 26 tables that include patient demographics such as age, sex, ethnicity, diagnoses, vital signs, and laboratory data. All data can be extracted using PostgreSQL software (version 14.0, PostgreSQL Global Development Group, Santa Barbara, California, USA). Detailed information on database utilization could be found in the MIMIC Online Documentation (https://mimic.mit.edu/). One author (X.T.) obtained access to the database and was responsible for data extraction (certification number 43334826).

Sepsis was clinically defined using the current Sepsis-3 diagnostic criteria [16]. Patients with suspected or confirmed infection who were administered antibiotics and had microbiological cultures of bodily fluids and a sequential organ failure assessment (SOFA) score ≥ 2 were enrolled in this study. The primary objects were patients who received multiple vasopressor agents, including NE, epinephrine, phenylephrine, dopamine, or vasopressin, within 24 h after the first vasopressor administration. The primary exposure was the timing of the administration of the vasopressors, which was based on the last dose of NE. The SSC recommends adding a second vasopressor when patients have an inadequate mean BP after the administration of 0.25−0.5 μg/kg/min of NE [1]. The participants were divided into early (last NE dose ≤ 0.25 μg/kg/min) and normal (last NE dose > 0.25 μg/kg/min) groups. For patients who received multiple types of vasopressors, the last NE dose was set to 0 μg/kg/min. All patients included in the study were aged 18–90 years and were admitted to the intensive care unit (ICU) for the first time. The primary outcome was 28-day mortality. The secondary outcomes were in-hospital mortality, ICU mortality, and acute kidney injury (AKI) within 7 days after vasopressor administration.

### Data extraction

Data extraction was performed using PostgreSQL 14.5 (PostgreSQL Global Development Group, Santa Barbara, California, USA). First, parameters related to the use of vasopressors, including maximum dose within 24 h (NE-equivalent doses were used to unify the different vasopressors’ dosages [17]) and interval from hypotension episode (defined as a systolic BP < 90 mmHg or a mean arterial pressure < 70 mmHg within 24 h before vasopressor administration) to NE administration, were extracted. The patient’s baseline characteristics (age, sex, ethnicity, and first care unit), interventions (antibiotic administration within 1 h after sepsis, renal replacement therapy, and mechanical ventilation), scoring system (SOFA, Charlson Comorbidity Index [CCI]), comorbidities (endocarditis, coronary atherothrombotic disease, atrial fibrillation, congestive heart failure, chronic obstructive pulmonary disease, stroke, chronic renal disease, liver disease, malignant tumor, respiratory failure, acute respiratory distress syndrome, and pneumonia), laboratory results (white blood cell count, hemoglobin, blood urea nitrogen, prothrombin time, activated partial thromboplastin time, international normalized ratio, bicarbonate, anion gap, sodium, chloride, potassium, lactate), and vital signs (systolic BP, diastolic BP, mean arterial pressure [MAP], heart rate, respiratory rate, temperature [°C], and oxygen saturation) were also recorded. Only measurements obtained within 24 h after the first vasopressor administration were extracted. The mean value was used when multiple measurements were available. Laboratory indicators included in the SOFA score were no longer compared. All comorbidities were collected based on the International Classification of Diseases, 9th and 10th Edition codes.

### Statistical analysis

Continuous variables are presented as mean and standard deviation or median and interquartile range (IQR). Categorical variables are presented as a total number and percentage. The Shapiro–Wilk test was used for data normality. The t-test and Mann–Whitney U test were used to compare continuous variables, while the chi-square test and Fisher exact test were used to compare categorical variables. Multiple imputations (MIs) were used when the data were missing (see Additional file 1: Table S1). The outcomes of patients in the early and normal groups were compared using a multivariate Cox proportional hazards regression model. Covariates with a p-value < 0.1 in the univariate analysis were included in the final multivariable analysis as potential confounders. The multivariate Cox proportional hazard models were fitted to each of the five datasets, and the results were pooled using the combining rules of MI. We also used stabilized inverse probability treatment weighting (sIPTW) and propensity score matching (PSM) to evaluate the consistency of the results.

Logistic regression analysis was used to estimate the patients’ PS to minimize covariate imbalance. Variables that were significantly different between the groups (standardized mean difference > 0.1) or those that appear to influence the outcomes, including interval from hypotension episode to NE administration, admission type, maximum NEQ dose, antibiotic use < 1 h from sepsis, gender, race, first ICU admission, SOFA score, renal replacement therapy, congestive heart failure, atrial fibrillation, coronary atherothrombotic disease, malignancy, pneumonia, heart rate, systolic BP, MAP, respiratory rate, oxygen saturation, prothrombin time, activated partial thromboplastin time, international normalized ratio, anion gap, and bicarbonate, blood urea nitrogen, sodium, chloride, potassium, and lactate levels, were considered as candidate variables in the PS calculation. The survival differences between the groups were illustrated using Kaplan–Meier survival curves. Restricted cubic spline (RCS) curves, fitted for the same multivariate Cox proportional hazards with three knots at the 10th, 50th, and 90th percentiles of the NE dose when adding a second vasopressor, were used to illustrate the association. All statistical analyses were conducted using R software (version 4.1.0, available at http://www.R-project.org).

## Results

**Fig. 1:**
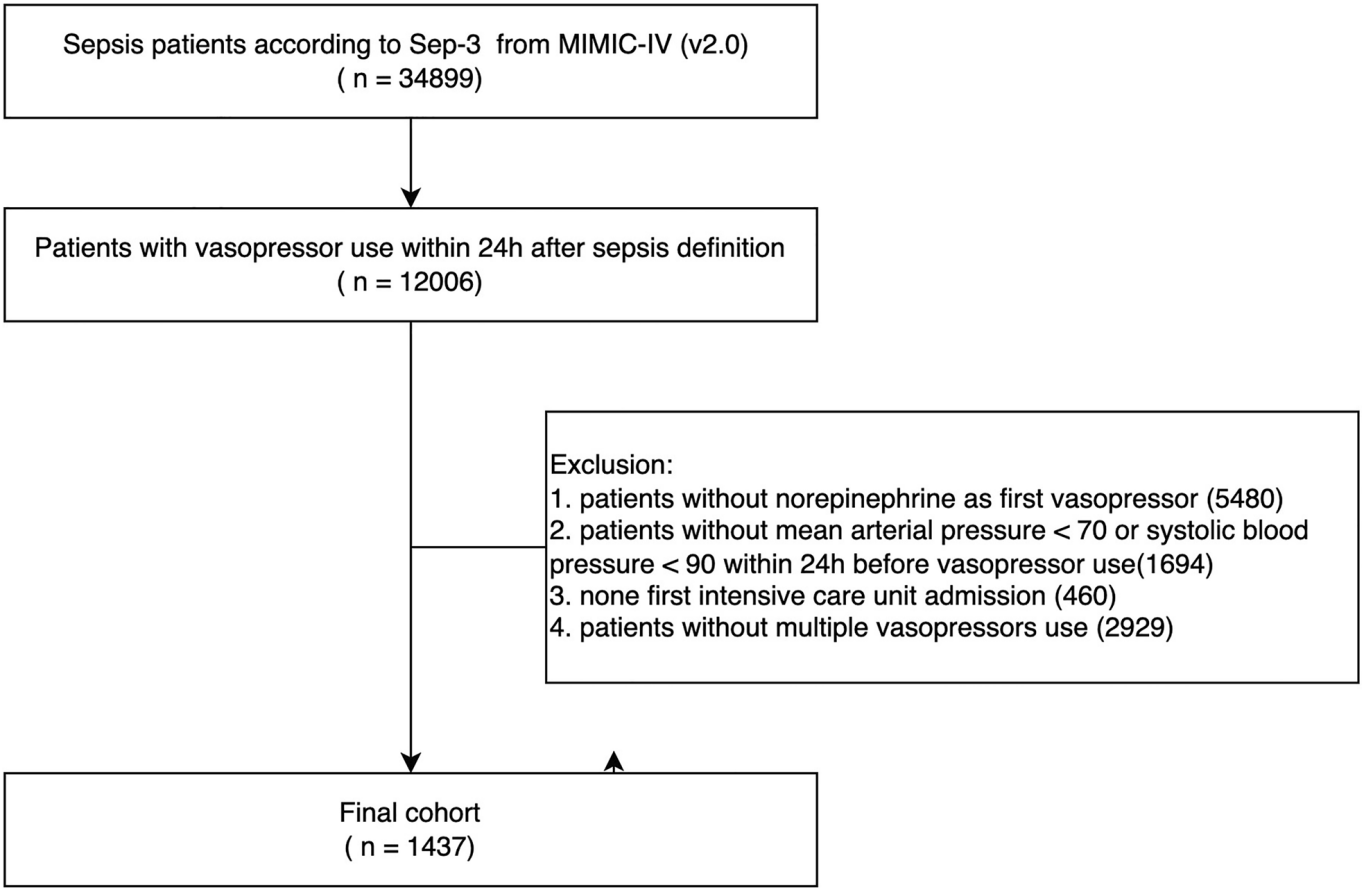
Flow chart of patient inclusion

Overall, 1437 patients were included in the study (Fig. 1). The 28-day mortality rate was 49.1% (Table 1). The median patient age was 68.2 (IQR, 57.4–78.7) years; 894 (59.1%) were men. The median interval from the first hypotension episode to NE administration was 6 (IQR, 0–58) minutes. The median maximum NEQ dose of total vasopressors was 0.88 (IQR, 0.52–1.26) μg/kg/min. The median last NE dose was 0.18 (IQR, 0–0.35) μg/kg/min. Using a cutoff of 0.25 μg/kg/min for the last NE dose, the early group included 851 patients (median last NE dose, 0 [IQR, 0–0.12] μg/kg/min), and the normal group included 586 patients (median last NE dose, 0.40 [IQR, 0.30–0.50] μg/kg/min). Compared with patients in the early group, those in the normal group had higher SOFA scores (13 [IQR, 11–16] vs. 12 [IQR, 9–14], p < 0.001) and lower MAPs (68.99 [IQR, 64.20–73.98] vs. 70.08 [IQR, 65.91–74.40], p = 0.005).

**Table 1:** Patient demographics

	Early group (n = 851)	Normal group (n = 586)	SMD	Early group (n = 861)	Normal group (n = 558)	SMD
Age (median [IQR])	69.1 [58.0, 78.8]	67.0 [56.7, 78.3]	0.048	67.9 [56.8, 78.0]	67.67 [57.27, 79.24]	0.078
Male (%)	522 (61.3)	327 (55.8)	0.113	508 (59.0)	327 (58.6)	0.009
Weight (median [IQR])	81.0 [69.6, 97.5]	78.8 [65.6, 97.5]	0.076	80.6 [68.7, 96.8]	78.20 [65.09, 97.16]	0.070
Race (%)			0.165			0.033
White	19 (2.2)	23 (3.9)		26 (3.1)	18 (3.2)	
Black	61 (7.2)	62 (10.6)		72 (8.4)	48 (8.7)	
Hispanic	30 (3.5)	21 (3.6)		31 (3.6)	21 (3.7)	
Asian	176 (20.7)	123 (21.0)		185 (21.5)	125 (22.4)	
Other	565 (66.4)	357 (60.9)		547 (63.5)	346 (62.0)	
Admission type (%)			0.200			0.086
Elective	192 (22.6)	93 (15.9)		168 (19.5)	94 (16.7)	
Emergency	449 (52.8)	362 (61.8)		491 (57.1)	341 (61.0)	
Urgent	210 (24.7)	131 (22.4)		202 (23.4)	124 (22.3)	
First care unit (%)			0.586			0.095
Cardiac ICU	281 (33.0)	63 (10.8)		203 (23.6)	110 (19.7)	
Medical/Surgical ICU	382 (44.9)	391 (66.7)		465 (54.1)	318 (56.9)	
Neuro ICU	6 (0.7)	8 (1.4)		9 (1.0)	6 (1.1)	
TSICU	182 (21.4)	124 (21.2)		184 (21.4)	124 (22.3)	
Time from hypotension episode to NE use (minute, median [IQR])	3.0 [0.0, 24.5]	13.0 [0.0, 147.8]	0.174	5.0 [0.0, 35.7]	10.00 [0.00, 105.80]	0.02
Maximum NEQ dose (median [IQR])	0.64 [0.37, 1.15]	1.06 [0.82, 1.35]	0.201	0.77 [0.4, 1.3]	1.02 [0.80, 1.30]	0.036
Scoring system						
SOFA (median [IQR])	12 [9, 14]	13 [11, 16]	0.372	12 [10, 15]	12.00 [10.00, 15.00]	0.029
Charlson comorbidity index (median [IQR])	6 [4, 8]	6 [5, 8]	0.057	6 [4, 8]	6.00 [5.00, 8.00]	0.024
Intervention						
Antibiotic use < 1 h from sepsis (%)	334 (39.2)	265 (45.2)	0.121	356 (41.3)	238 (42.5)	0.025
RRT (%)	128 (15.0)	131 (22.4)	0.188	157 (18.2)	103 (18.4)	0.004
MV (%)	685 (80.5)	477 (81.4)	0.023	697 (81.0)	457 (81.8)	0.022
Comorbidity						
Endocarditis (%)	18 (2.1)	13 (2.2)	0.007	20 (2.4)	15 (2.7)	0.024
CHF (%)	331 (38.9)	186 (31.7)	0.15	312 (36.2)	192 (34.4)	0.037
AFIB (%)	364 (42.8)	181 (30.9)	0.248	323 (37.5)	1978 (35.4)	0.044
Renal disease (%)	219 (25.7)	159 (27.1)	0.032	219 (25.5)	149 (26.6)	0.026
Liver disease (%)	95 (11.2)	84 (14.3)	0.095	116 (13.5)	73 (13.1)	0.013
COPD (%)	144 (16.9)	81 (13.8)	0.086	141 (16.3)	81 (14.5)	0.051
CAD (%)	287 (33.7)	123 (21.0)	0.289	240 (27.8)	144 (25.7)	0.048
Stroke (%)	44 (5.2)	26 (4.4)	0.034	42 (4.8)	37 (6.2)	0.059
Malignancy (%)	149 (17.5)	146 (24.9)	0.182	177 (20.6)	121 (21.6)	0.025
Respiratory failure (%)	389 (45.7)	296 (50.5)	0.096	420 (48.8)	282 (50.6)	0.034
ARDS (%)	72 (8.5)	60 (10.2)	0.061	76 (8.8)	58 (10.3)	0.051
Pneumonia (%)	286 (33.6)	230 (39.2)	0.117	313 (36.4)	214 (38.4)	0.041
Vital sign						
Heart rate (median [IQR])	92.85 [80.14, 107.10]	97.35 [84.15, 109.53]	0.175	96.38 [81.55, 108.91]	96.66 [84.48, 108.01]	0.044
Systolic BP (median [IQR])	103.79 [97.78, 110.40]	101.98 [95.86, 108.14]	0.177	102.51 [96.67, 109.60]	102.24 [96.26, 108.25]	0.034
Diastolic BP (median [IQR])	55.98 [50.56, 61.48]	55.39 [49.59, 60.69]	0.052	55.81 [50.38, 61.47]	55.84 [49.96, 60.79]	0.004
MAP (median [IQR])	70.08 [65.91, 74.40]	68.99 [64.20, 73.98]	0.143	69.61 [65.25, 74.17]	69.40 [64.79, 74.51]	0.005
Respiratory rate (median [IQR])	21.00 [18.23, 24.48]	23.08 [19.79, 26.06]	0.364	21.83 [18.88, 25.38]	22.31 [19.38, 25.55]	0.064
Temperature (median [IQR])	36.84 [36.50, 37.22]	36.85 [36.45, 37.36]	0.037	36.84 [36.45, 37.28]	36.82 [36.43, 37.34]	0.011
Oxygen saturation (median [IQR])	97.17 [95.28, 98.43]	96.43 [94.21, 98.07]	0.145	96.95 [94.84, 98.30]	96.70 [94.50, 98.20]	0.037
Laboratory indicator						
WBC (median [IQR])	14.45 [9.90, 20.02]	15.37 [8.87, 21.85]	0.086	14.55 [9.60, 20.68]	15.49 [9.10, 21.85]	0.041
Hemoglobin (median [IQR])	9.80 [8.84, 11.23]	9.96 [8.70, 11.58]	0.036	9.80 [8.75, 11.35]	9.99 [8.77, 11.62]	0.049
BUN (median [IQR])	29.40 [19.00, 45.58]	37.33 [23.04, 55.85]	0.261	31.90 [20.00, 50.00]	34.00 [21.00, 53.33]	0.037
PT (median [IQR])	16.82 [14.38, 21.75]	18.30 [14.65, 24.71]	0.181	17.25 [14.60, 23.13]	17.71 [14.45, 23.44]	0.029
APTT (median [IQR])	37.20 [31.19, 48.52]	40.22 [32.34, 55.06]	0.158	38.26 [31.45, 51.24]	39.87 [31.80, 53.96]	0.038
INR (median [IQR])	1.53 [1.30, 2.03]	1.69 [1.35, 2.30]	0.167	1.60 [1.30, 2.15]	1.62 [1.30, 2.15]	0.027
Bicarbonate (median [IQR])	19.50 [16.00, 22.50]	17.17 [14.00, 20.58]	0.400	18.50 [15.00, 21.67]	18.00 [15.00, 21.50]	0.034
Anion gap (median [IQR])	16.25 [13.00, 20.17]	18.82 [15.75, 23.32]	0.432	17.20 [14.00, 21.50]	17.60 [14.74, 22.00]	0.043
Sodium (median [IQR])	138.00 [135.00, 141.00]	137.50 [134.00, 140.50]	0.126	137.80 [134.50, 140.80]	137.64 [134.00, 140.67]	0.008
Chloride (median [IQR])	105.00 [100.50, 109.00]	103.33 [98.00, 107.75]	0.216	104.33 [99.50, 108.50]	104.00 [99.00, 108.33]	0.019
Potassium (median [IQR])	4.33 [3.95, 4.75]	4.40 [4.00, 5.04]	0.193	4.37 [3.95, 4.86]	4.34 [3.95, 4.93]	0.001
Lactate (median [IQR])	3.13 [1.90, 5.29]	3.96 [2.40, 6.64]	0.252	3.35 [2.00, 5.92]	3.66 [2.23, 6.11]	0.015

*SMD* standardized mean difference, *IQR* inter quartile range, *ICU* intensive care unit, *NEQ* norepinephrine-equivalent doses, *SOFA* sequential organ failure assessment, *RRT* renal replacement therapy, *MV* mechanical ventilation, *CHF* congestive heart failure, *AFIB* atrial fibrillation, *COPD* chronic obstructive pulmonary disease, *CAD* coronary atherothrombotic disease; ARDS, acute respiratory distress syndrome, *BP* blood pressure, *MAP* mean artery pressure, *WBC* white blood cell; *BUN* blood urea nitrogen, *PT* prothrombin time, *APTT* activated partial thromboplastin time, *INR* international normalized ratio

**Fig. 2:**
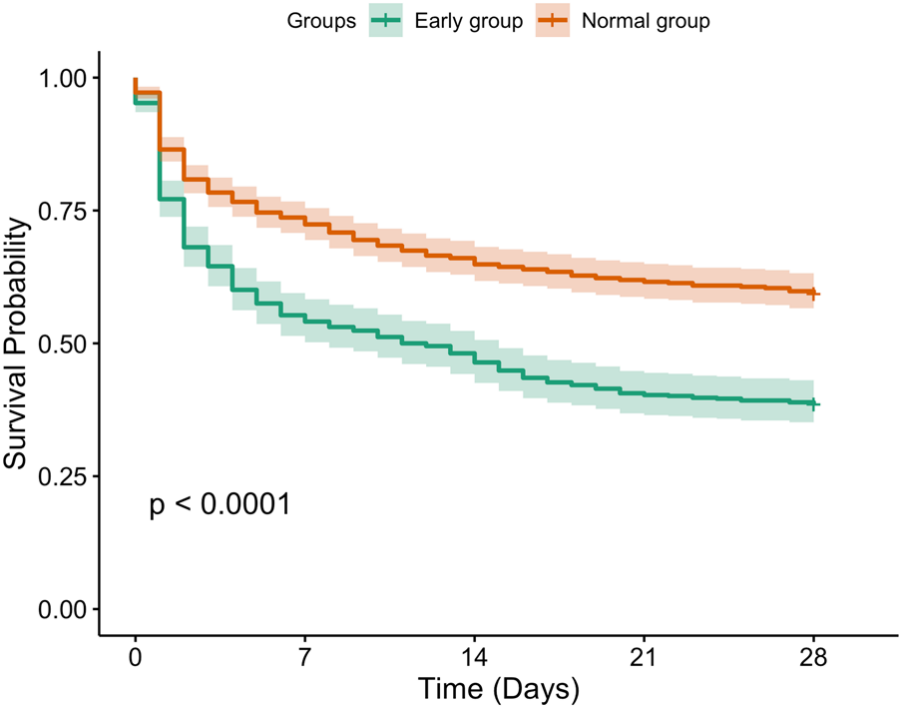
Kaplan–Meier curves of 28-day overall survival. The 28-day overall survival of patients in the early and normal groups

**Fig. 3:**
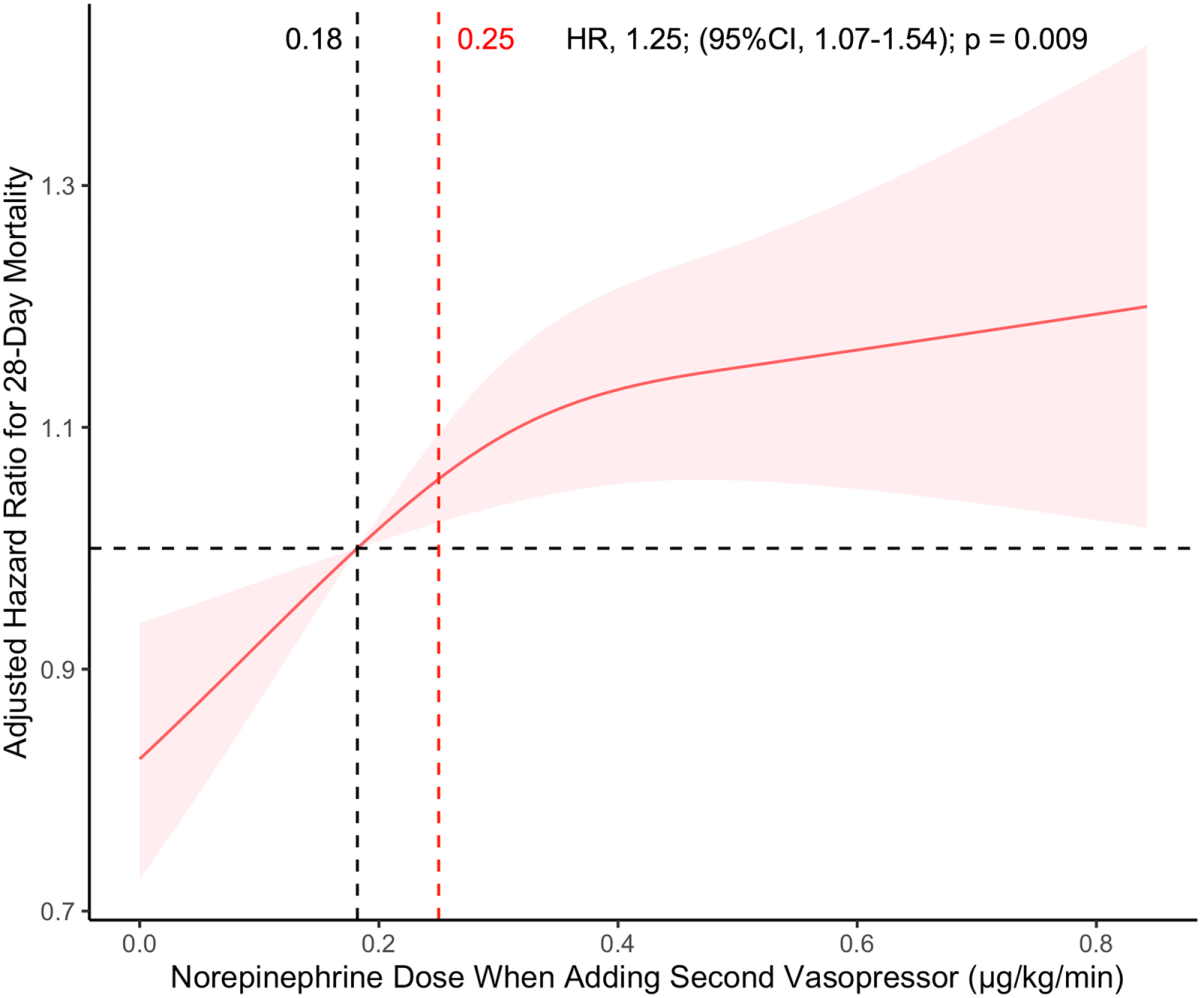
Restricted cubic spline curve based on the multivariate Cox proportional hazards models. The multivariable-adjusted hazard ratios for 28-day mortality based on the dose of the initial vasopressor when additional vasopressors were administered are shown on a continuous scale. The solid red lines represent the multivariable-adjusted hazard ratios, and the pink filling represents the 95% confidence intervals derived from restricted cubic spline regressions with three knots. The dashed black lines show a hazard ratio of 1.0, indicating no association. The dashed red curves highlight the range of 0.25 μg/kg/min, which is suggested by the Surviving Sepsis Campaign for the administration of additional vasopressors

The multivariable analysis was adjusted using the maximum NEQ dose, admission type, antibiotic use < 1 h from sepsis, gender, weight, race, age, first ICU admission, SOFA, CCI, renal replacement therapy, congestive heart failure, atrial fibrillation, renal, liver, and coronary diseases, respiratory failure, heart rate, systolic BP, diastolic BP, MAP, respiratory rate, temperature, oxygen saturation, prothrombin time, activated partial thromboplastin time, international normalized ratio, and bicarbonate, blood urea nitrogen, sodium, chloride, potassium, and lactate levels. In the MI cohort, patients in the early group had a lower 28-day mortality (HR: 0.76; 95% CI: 0.65–0.89; p < 0.001) than patients in the normal group. Table 2 shows that the results in the PSM (HR: 0.80; 95% CI: 0.68–0.94; p = 0.006) and sIPTW (HR: 0.75; 95% CI: 0.63–0.88; p < 0.001) cohorts were similar to those in the original MI cohort (before PSM and sIPTW) (Fig. 2). These results showed that patients in whom multiple vasopressors are immediately initiated have lower 28-day mortality. RCS curves showed that the risk of 28-day mortality increased as the last NE dose increased (Fig. 3). The assessed HR of 1.0 was at 0.18 μg/kg/min of the last NE dose (Fig. 3). After sIPTW, patients in the early group had significantly lower in-hospital and ICU mortality. However, no difference was found in the incidence of AKI within 7 days after vasopressor administration (Table 3).

**Table 2:** Association between last norepinephrine dose and the 28-day mortality in the early and normal groups

	Hazard ratio	95% Confidence interval	p-value
MI cohort	0.76	0.65–0.89	< 0.001
PSM cohort	0.80	0.68–0.94	0.006
sIPTW cohort	0.75	0.63–0.88	< 0.001

*MI* multiple imputation, *PSM* propensity score matching, *sIPTW* stabilized inverse probability treatment weighting

**Table 3:** Outcomes in the stabilized inverse probability treatment weighting cohorts

Outcomes	Early group (%)	Normal group (%)	p-value
28-day mortality	46.5	58.0	< 0.001
In-hospital mortality	43.6	56.5	< 0.001
ICU mortality	35.6	42.6	0.016
AKI in 7 days after vasopressor use	75.6	76.4	0.744

*ICU* intensive care unit, *AKI* acute kidney injury

## Discussion

In this study, the timing of the administration of multiple vasopressors was significantly associated with outcomes in patients with sepsis. Early multiple vasopressor administration was strongly associated with a lower risk-adjusted 28-day mortality.

Hemodynamic support to maintain sufficient perfusion pressure and oxygen supply to the capillaries and organs is important in patients with septic shock. Prompt perfusion pressure reversal is crucial [18]. Traditionally, a stepwise method is used, suggesting that vasopressor support is a rescue therapy after the failure of initial fluid resuscitation to correct hypotension or when the perfusion pressure is insufficient to maintain proper tissue perfusion. However, the stepwise method may delay treatment and prolong hypotension, which has been associated with mortality in patients with shock [4, 19, 20]. Therefore, several methods to maintain the perfusion pressure early in a patient’s disease course have been reported.

Early administration of a vasopressor can be used to maintain perfusion pressure. Early vasopressor administration in patients with septic shock is related to lower mortality rates [7, 18, 21–24]. The administration of a one-hour bundle to patients with life-threatening hypotension to maintain a MAP ≥ 65 mmHg following failed fluid resuscitation has been proposed by the SSC [1]. Rapid-start vasopressor treatment reduces the severity and duration of hypotension, enhances cardiac output, improves coronary artery and microcirculatory perfusion, improves MAP, and reverses severe hypotension [11].

Early administration of multimodal vasopressors may be considered a physiologically-guided approach to the complex, refractory, and multifactorial pathogenesis of septic shock [5, 12]. However, there are no data that support this hypothesis; therefore, the current exploratory analysis of the MIMIC-IV database was conducted. Patients who received additional vasopressors within 24 h of the first vasopressor administration were included in this analysis, while patients with other types of shock or conditions were excluded to avoid bias. The administration of two or more vasopressors within a short period may indicate refractory septic shock. The multivariate analysis showed that early administration of more than one vasopressor was strongly associated with lower in-hospital mortality rates; the results in the MI, PSM, and sIPTW cohorts were similar. These results support the early administration of multimodal vasopressors [5].

Early administration of multimodal vasopressors may have several benefits. First, different vasopressors have complementary mechanisms of action [25]. For example, NE has high vasopressor potency and increases the cardiac index without increasing the heart rate or myocardial oxygen consumption [26], dopamine increases cardiac contractility and stroke volume while augmenting renal perfusion and urinary output [27], and vasopressin increases urinary output and improves creatine clearance [28, 29]. Second, early administration may reduce the required dosage, especially during the initiation stage of vasopressin [5]. Sacha et al. reported that a higher NEQ dose during vasopressin initiation is associated with higher in-hospital mortality in patients with septic shock [30]. Third, changes in the host genotype [31–33], varying organ-specific receptor expressions, and downregulation of distinct tissues [34] may result in a heterogeneous response to different types of vasopressors, which may be mitigated by early administration of multimodal vasopressors. Fourth, patients who respond to vasopressors have better outcomes than those who do not [12, 17, 35], highlighting the fact that treatment sensitivity should be addressed during vasopressor selection. Early administration of multimodal vasopressors may help assess a patient’s sensitivity to vasopressors [12].

Early administration of multimodal vasopressors is challenging. First, the complementary actions of the hemodynamic support of different types of vasopressors may be accompanied by complementary adverse effects. Second, early administration may not be cost-effective and may result in increased drug resistance or overdose. Finally, patients with benign shock who require a small amount of catecholamines to correct hypotension do not require the use of several drugs. Hence, the timing of initial vasopressor failure and the necessity of more than one vasopressor remain unclear.

This study has several limitations. Identifying patients with refractory septic shock in the MIMIC-IV database was challenging. This cohort may include misclassified patients with septic shock or other types of shock. The major purpose of this study was to evaluate the timing of the simultaneous use of several vasopressors; therefore, selecting all patients who received at least two types of vasopressors within 24 h may be appropriate. Second, certain critical information, such as the reason used to choose the first or second vasopressor or the protocols used, was unavailable. Third, this study is an exploratory analysis of data in the MIMIC-IV database. Due to the retrospective nature of the study, generalization of conclusions needs to be done with caution. More studies should be conducted to further investigate the advantages and disadvantages of the early administration of multimodal vasopressors.

## Conclusion

The timing of administration of secondary vasopressors is strongly related to the outcomes of patients with sepsis who receive at least two types of vasopressors. Patients with sepsis in whom a second vasopressor is administered after receiving an NE dose of < 0.25 μg/kg/min have a lower adjusted risk of 28-day mortality. Future prospective studies are needed to further examine the relationship between the timing of administration of secondary vasopressors and patient outcomes.

## Supplementary Information

**Additional file 1: Table S1.** Missing value.

## Data Availability

The datasets presented in the current study are available in the MIMIC-IV database (https://physionet.org/content/mimiciv/2.0/).

## Abbreviations

sIPTW: Stabilized inverse probability of treatment weighting
RCS: Restricted cubic spline
BP: Blood pressure
NE: Norepinephrine
SSC: Surviving sepsis campaign
SOFA: Sequential organ failure assessment
ICU: Intensive care unit
AKI: Acute kidney injury
CCI: Charlson comorbidity index
MAP: Mean arterial pressure
IQR: Interquartile range
MI: Multiple imputation
