# Supplementary material for: Early administration of multiple vasopressors is associated with better survival in patients with sepsis: a propensity score-weighted study
Source: Eur J Med Res. 2023 Jul 22;28:249. doi: 10.1186/s40001-023-01229-w (PMC10362716; doi:10.1186/s40001-023-01229-w)
Supplement: Supplementary file 1 — Additional file 1: Table S1. Missing value. [file 40001_2023_1229_MOESM1_ESM.docx]

Additional file 1: Table S1. Missing value.

| variable | missing | complete | percent_complete | percent_missing |
| --- | --- | --- | --- | --- |
| Temperature | 133 | 1304 | 90.7446068 | 9.2553932 |
| Lactate | 60 | 1377 | 95.8246347 | 4.1753653 |
| INT | 47 | 1390 | 96.7292971 | 3.2707029 |
| PT | 46 | 1391 | 96.7988866 | 3.2011134 |
| APTT | 45 | 1392 | 96.868476 | 3.131524 |
| Diastolic BP | 12 | 1425 | 99.1649269 | 0.8350731 |
| Systolic BP | 12 | 1425 | 99.1649269 | 0.8350731 |
| Hemoglobin | 8 | 1429 | 99.4432846 | 0.5567154 |
| WBC | 7 | 1430 | 99.512874 | 0.487126 |
| Anion gap | 5 | 1432 | 99.6520529 | 0.3479471 |
| Bicarbonate | 4 | 1433 | 99.7216423 | 0.2783577 |
| BUN | 4 | 1433 | 99.7216423 | 0.2783577 |
| Chloride | 4 | 1433 | 99.7216423 | 0.2783577 |
| Potassium | 4 | 1433 | 99.7216423 | 0.2783577 |
| Sodium | 4 | 1433 | 99.7216423 | 0.2783577 |
| Oxygen saturation | 4 | 1433 | 99.7216423 | 0.2783577 |
| weight | 3 | 1434 | 99.7912317 | 0.2087683 |
